# RNA sequencing-based analysis of the magnum tissues revealed the novel genes and biological pathways involved in the egg-white formation in the laying hen

**DOI:** 10.1186/s12864-021-07634-x

**Published:** 2021-05-01

**Authors:** Nirvay Sah, Donna Lee Kuehu, Vedbar Singh Khadka, Youping Deng, Rajesh Jha, Sanjeev Wasti, Birendra Mishra

**Affiliations:** 1 Department of Human Nutrition, Food and Animal Sciences, University of Hawaii at Manoa, HI 96822 Honolulu, USA; 2 Department of Molecular Biosciences and Bioengineering, University of Hawaii at Manoa, Honolulu, HI 96822 USA; 3 Department of Quantitative Health Sciences, John A. Burns School of Medicine, University of Hawaii at Manoa, Honolulu, HI 96813 USA

**Keywords:** Egg formation, Egg-white, Laying hens, Magnum, RNA-Seq, Transcriptome

## Abstract

**Background:**

The mechanism of egg formation in the oviduct of laying hens is tightly controlled; each segment of the oviduct contributes a unique component of the egg. Several genes/proteins are involved in the synthesis of a completely healthy egg. This implies a time- and tissue-specific expression of genes and proteins in the different oviductal segments. We used hens at different physiological stages and time points to understand the transcriptional regulation of egg-white (albumen) synthesis and secretion onto the eggs in the magnum of laying hens. This study used Next-Generation Sequencing and quantitative real-time PCR (qPCR) to detect the novel genes and the cognate biological pathways that regulate the major events during the albumen formation.

**Results:**

Magnum tissues collected from laying (*n* = 5 each at 3 h post-ovulation, p.o. and 15–20 h p.o.), non-laying (*n* = 4), and molting (*n* = 5) hens were used for differential gene expression analyses. A total of 540 genes (152 upregulated and 388 down-regulated) were differentially expressed at 3 h p.o. in the magnum of laying hens. Kyoto Encyclopedia of Genes and Genomes pathways analysis of the 152 upregulated genes revealed that glycine, serine, and threonine metabolism was the most-enriched biological pathway. Furthermore, the top two most enriched keywords for the upregulated genes were amino-acid biosynthesis and proteases. Nine candidate genes associated with albumen formation were validated with qPCR to have differential expression in laying, non-laying, and molting hens. Proteases such as *TMPRSS9*, *CAPN2*, *MMP1*, and *MMP9* (protein maturation, ECM degradation, and angiogenesis); enzymes such as *PSPH*, *PHGDH*, and *PSAT1* (amino-acid biosynthesis); *RLN3*, *ACE,* and *REN* (albumen synthesis, secretion and egg transport); and *AVD*, *AvBD11*, and *GPX3* (antimicrobial and antioxidants) were recognized as essential molecules linked to albumen deposition in the magnum.

**Conclusions:**

This study revealed some novel genes that participate in the signaling pathways for egg-white synthesis and secretion along with some well-known functional genes. These findings help to understand the mechanisms involved in albumen biosynthesis.

**Supplementary Information:**

The online version contains supplementary material available at 10.1186/s12864-021-07634-x.

## Background

The chicken oviduct is a long tubular organ with histologically and functionally five distinct segments (infundibulum, magnum, isthmus, shell gland, and vagina) having specific functions in egg formation. The ovulated egg-yolk traverses through the magnum in about 2–3 h, during which the egg-white (albumen) is continuously deposited around it. Though 88% of the albumen is water, this component of the egg contributes more than 60% to the total egg weight and determines the quality of an egg. Smaller eggs cannot make it to the hatcheries and also have low market-value as table eggs. Consumers consider egg as a “functional food” because of several proteins incorporated in the albumen [1]. Fundamentally, the albumen is the primary source of nutrients and a barrier to the pathogenic infections of the developing embryo [2]. The food processing industry uses only the albumen portion of the egg for its foaming and gelling properties [3]; These perspectives necessitate an egg with qualitative and proportionate albumen in it.

The synthesis and storage of the principle egg-white proteins (i.e., ovalbumin, ovotransferrin, ovomucoid, and lysozyme), constituting approximately 90% of the total albumen protein, occur exclusively in the tubular gland cells of the magnum epithelium [4, 5]. Following the exit of an egg from the magnum, the epithelial cells of the magnum begin the synthesis and storage of the egg-white proteins to be deposited around the next egg, which continues for 20–23 h [6, 7]. Each egg-white protein is synthesized at a rate proportional to its composition in the egg-white [5]. After synthesis, the essential proteins are packaged in secretory granules and secreted from the tubular glands into the lumen of the magnum, where they are deposited over the egg yolk [5, 8]. The cellular signaling for the biosynthesis of albumen is regulated by estrogen, progesterone, and testosterone [5, 8]. The existence of an egg in the magnum causing a mechanical distention of the magnum wall, which stimulates the secretion of the stored egg-white proteins [8, 9]. Some transcriptomic studies and some gene-specific studies have highlighted the importance of several genes/proteins in albumen synthesis and secretion [10–13]. The solute carriers, a large family of membrane transporters, transport glucose, amino acids, and electrolytes across the magnum epithelium, are upregulated during the egg-white formation [14]. The matrix metalloproteases rapidly degrade the collagens and other matrix proteins underlying the cells of the magnum for continuous cellular growth and development [15]. Also, proteins incorporated during the egg-white formation in the magnum further determine the structures of calcium crystals being formed on the eggshell during mineralization in the shell gland [16]. The egg-white is a composite of several proteins whose secretion and synthesis are very intricate, and their regulation in the oviduct is not clearly understood. Therefore, we hypothesized that the transcriptomic analyses, using RNA-Sequencing (RNA-Seq), of the magnum of laying hens in contrast to a magnum of non-laying hens can reveal the novel genes and biological pathways involved in the regulation of egg-white synthesis and secretion.

In this study, we analyzed the genes and cognate pathways active in the magnum of laying hens whose expressions are directly influenced by the presence of an egg. We further validated the expression profiles of novel genes in the laying (3 h, and 15–20 h post-ovulation, p.o.), molting, and non-laying hens.

## Results

### Identification of differentially expressed genes (DEGs) from RNA sequence

**Fig. 1:**
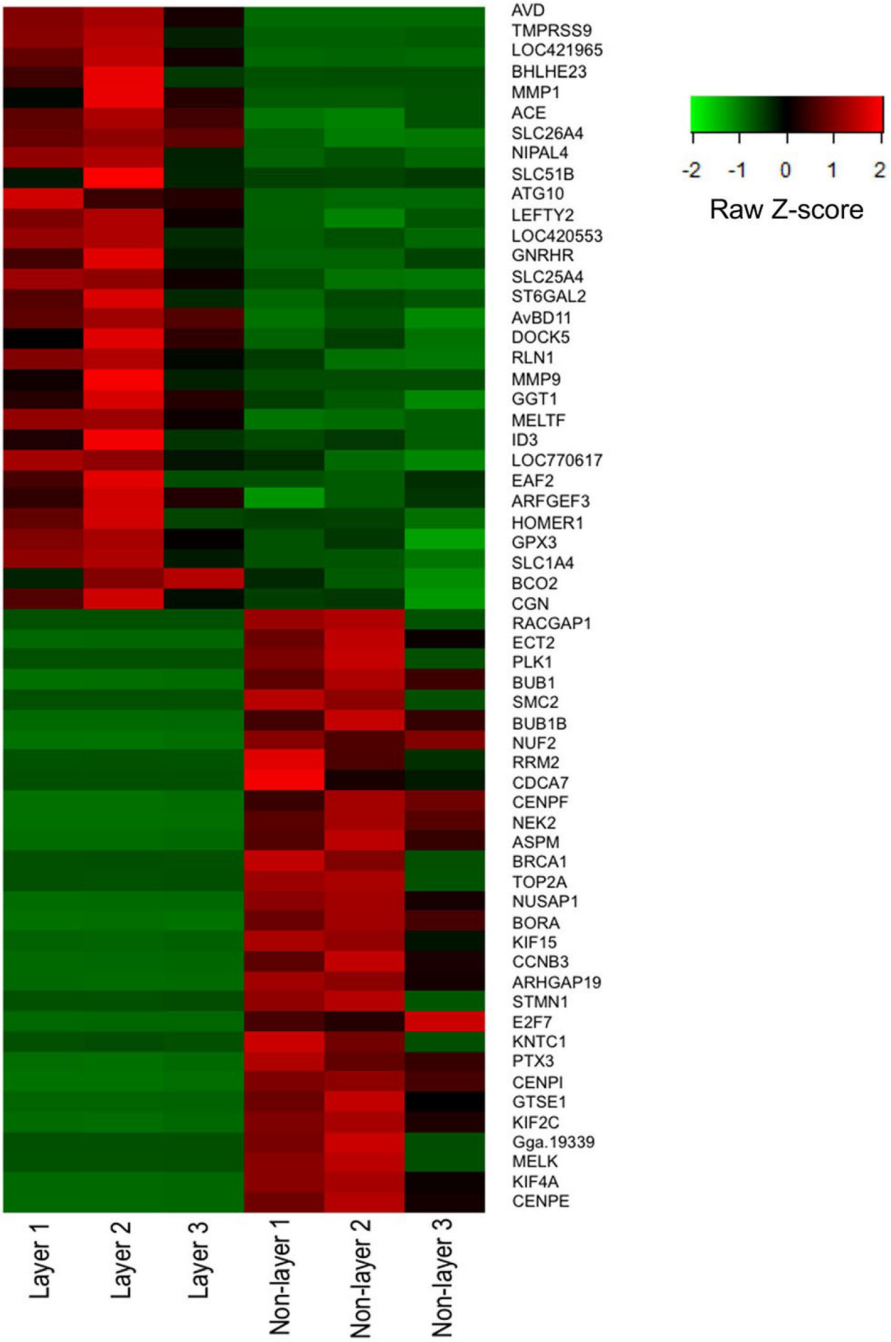
Heat map of top thirty DEGs in the magnum of laying and non-laying hens. The raw z-score depicts the standard deviation of the gene expression value from the mean after normalization. A gene having a negative z-score is represented by green color while a positive z-score is represented by red color. RNA-Seq was performed on magnum from three laying (3 h p.o.) and three non-laying hens. Transcripts were aligned to the chicken genome and mapped genes with at least a 3-fold change difference and Benjamini Hochberg q-value < 0.05 were considered differentially expressed

Raw sequencing reads in FASTQ format from replicated RNA-Seq libraries were obtained, and their qualities were checked using FastQC. There was an average of 30.5 M and 33.4 M original raw reads in laying and non-laying hens, respectively. After trimming and filtration, more than 97% of input reads from both laying and non-laying hens were found as excellent quality sequences (Supplementary Table S1). Mapping results to the chicken genome database showed that an average of 93.42% of the retained reads from layers and 87.88% from non-layers were uniquely mapped (Supplementary Table S2). A total of 19,152 transcripts were annotated from Ensembl alignment (release 94), representing 50.24% of the chicken genome assembly. The DESeq2 analysis showed that 540 genes were differentially expressed between laying and non-laying hens (comprehensive gene list in Supplementary Table S3). Among the differentially expressed genes (DEGs), 457 genes were officially characterized, while the rest were novel transcripts without any annotation. There were 152 upregulated and 388 downregulated genes in the magnum of laying hens (at 3 h p.o.) as compared to the non-laying hens. The top 30 upregulated and downregulated genes in the magnum of laying hens are presented in Tables 1 and 2, respectively. A visual representation of the 30 most upregulated and downregulated genes in layers is shown as a heatmap image (Fig. 1).

**Table 1:** The 30 most up-regulated DEGs in the magnum of laying compared to non-laying hens

Gene Name	Gene Description	Fold Change
*AVD*	*Avidin*	250.6824
*TMPRSS9*	*Transmembrane protease, serine 9*	33.0611
*SLC7A9*	*Solute carrier family 7 member 9*	29.9439
*BHLHE23*	*Basic helix-loop-helix family member e23*	17.2940
*MMP1*	*Matrix metallopeptidase 1*	16.0790
*ACE*	*Angiotensin converting enzyme*	14.8313
*SLC26A4*	*Solute carrier family 26 member 4*	9.7876
*NIPAL4*	*NIPA like domain containing 4*	8.8105
*SLC51B*	*Solute carrier family 51 beta subunit*	8.7190
*ATG10*	*Autophagy related 10*	8.6403
*LEFTY2*	*Left-right determination factor 1*	8.5433
*ACCSL*	*1-Aminocyclopropane-1-carboxylate synthase homolog (inactive) like*	8.4146
*GNRHR*	*Gonadotropin releasing hormone receptor*	7.7644
*SLC25A4*	*Solute carrier family 25 member 4*	7.5906
*ST6GAL2*	*ST6 beta-galactoside alpha-2,6-sialyltransferase 2*	7.5662
*AvBD11*	*Avian beta defensin 11*	7.5066
*DOCK5*	*Dedicator of cytokinesis 5*	7.4964
*RLN1*	*Relaxin*	7.4939
*MMP9*	*Matrix metallopeptidase 9*	7.4666
*GGT1*	*Gamma-glutamyltransferase 1*	7.0938
*MELTF*	*Melanotransferrin*	6.9221
*ID3*	*Inhibitor of DNA binding 1, HLH protein*	6.5950
*LOC770617*	*Transmembrane protein 100-like*	6.4296
*KIAA1109*	*Fragile site-associated protein*	6.4112
*EAF2*	*ELL associated factor 2*	6.0836
*ARFGEF3*	*ARFGEF family member 3*	5.8841
*HOMER1*	*Homer scaffolding protein 1*	5.6404
*GPX3*	*Glutathione peroxidase 3*	5.6046
*SLC1A4*	*Solute carrier family 1 member 4*	5.5812
*LOC419409*	*Golgi integral membrane protein 4-like*	5.4493

Transcripts from the magnum of layers and non-layers were aligned to the chicken genome and mapped genes with at least a 3-fold change difference and Benjamini Hochberg q-value < 0.05 were considered differentially expressed. DEGs, differentially expressed genes

**Table 2:** The 30 most down-regulated DEGs in the magnum of laying compared to non-laying hens

Gene Name	Gene Description	Fold Change
*RACGAP1*	*Rac GTPase activating protein 1*	16.6262
*ECT2*	*Epithelial cell transforming 2*	14.9228
*PLK1*	*Polo like kinase 1*	14.0534
*BUB1*	*BUB1 mitotic checkpoint serine/threonine kinase*	13.9712
*SMC2*	*Structural maintenance of chromosomes 2*	13.8266
*BUB1B*	*BUB1 mitotic checkpoint serine/threonine kinase B*	13.7985
*NUF2*	*NUF2, NDC80 kinetochore complex component*	13.3569
*RRM2*	*Ribonucleotide reductase regulatory subunit M2*	12.9268
*CDCA7*	*Cell division cycle associated 7*	12.8785
*CENPF*	*Centromere protein F*	12.7394
*NEK2*	*NIMA related kinase 2*	12.5734
*ASPM*	*Abnormal spindle microtubule assembly*	12.2945
*BRCA1*	*BRCA1(Breast cancer)*	12.2056
*TOP2A*	*Topoisomerase (DNA) II alpha*	11.5891
*NUSAP1*	*Nucleolar and spindle associated protein 1*	11.1474
*BORA*	*Bora, aurora kinase A activator*	11.0740
*KIF15*	*Kinesin family member 15*	10.6809
*CCNB3*	*CCNB3 (cyclin B3)*	10.6117
*ARHGAP19*	*ARHGAP19*	10.4602
*STMN1*	*Stathmin 1*	10.4540
*E2F7*	*E2F transcription factor 7*	10.4150
*KNTC1*	*Kinetochore associated 1*	10.0559
*PTX3*	*Pentraxin 3*	9.9658
*CENPI*	*Centromere protein I*	9.8662
*GTSE1*	*G2 and S-phase expressed 1*	9.7306
*KIF2C*	*Kinesin family member 2C*	9.6465
*Gga.19339*	*Family with sequence similarity 72, member A*	9.5760
*MELK*	*Maternal embryonic leucine zipper kinase*	9.4771
*KIF4A*	*Kinesin family member 4A*	9.2900
*CENPE*	*Centromere protein E*	9.2869

Transcripts from the magnum of layers and non-layers were aligned to the chicken genome and mapped genes with at least a 3-fold change difference and Benjamini Hochberg q-value < 0.05 were considered differentially expressed. DEGs, differentially expressed genes

### Functional annotation and pathways enrichment analysis of DEGs

**Fig. 2:**
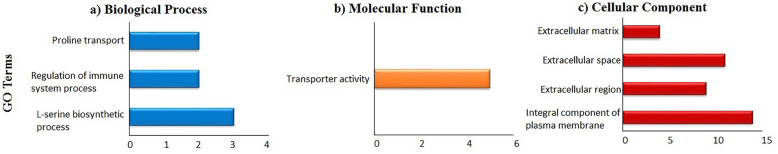
Gene Ontology enrichment analysis of upregulated genes in the magnum of laying and non-laying hens. **a** Biological Process, **b** Molecular Function, **c** Cellular Component. The up-regulated genes in the laying hens (3 h p.o.) were subjected to the DAVID database for Gene Ontology (GO) enrichment analysis. All the GO terms with a modified Fisher Exact p-value < 0.05 and a threshold gene count of 2 were considered enriched

The Database for Annotation, Visualization, and Integrated Discovery (DAVID) bioinformatics resource was used to gain insight into various Gene Ontology (GO) terms of the upregulated genes in layers. Only the annotated 121 genes that were upregulated in laying hens were uploaded for functional annotation in the DAVID system, and results showed 119 genes were annotated into the three GO terms; biological process, cellular component, and molecular function. Altogether 85 genes were recognized in the biological process, among which three processes; L-serine biosynthetic process, regulation of immune system process, and proline transport were enriched (Fig. 2a). The molecular function had only one enriched GO term, i.e., transporter activity, with 83 genes recognized (Fig. 2b), while the cellular component contained 4 enriched GO terms of the 90 identified genes (Fig. 2c). We also analyzed pathway enrichment for the upregulated genes in laying hens using the KEGG pathways as incorporated in the DAVID system. Glycine, serine, and threonine metabolism was the only pathway to be enriched for upregulated genes.

### Canonical pathways

**Fig. 3:**
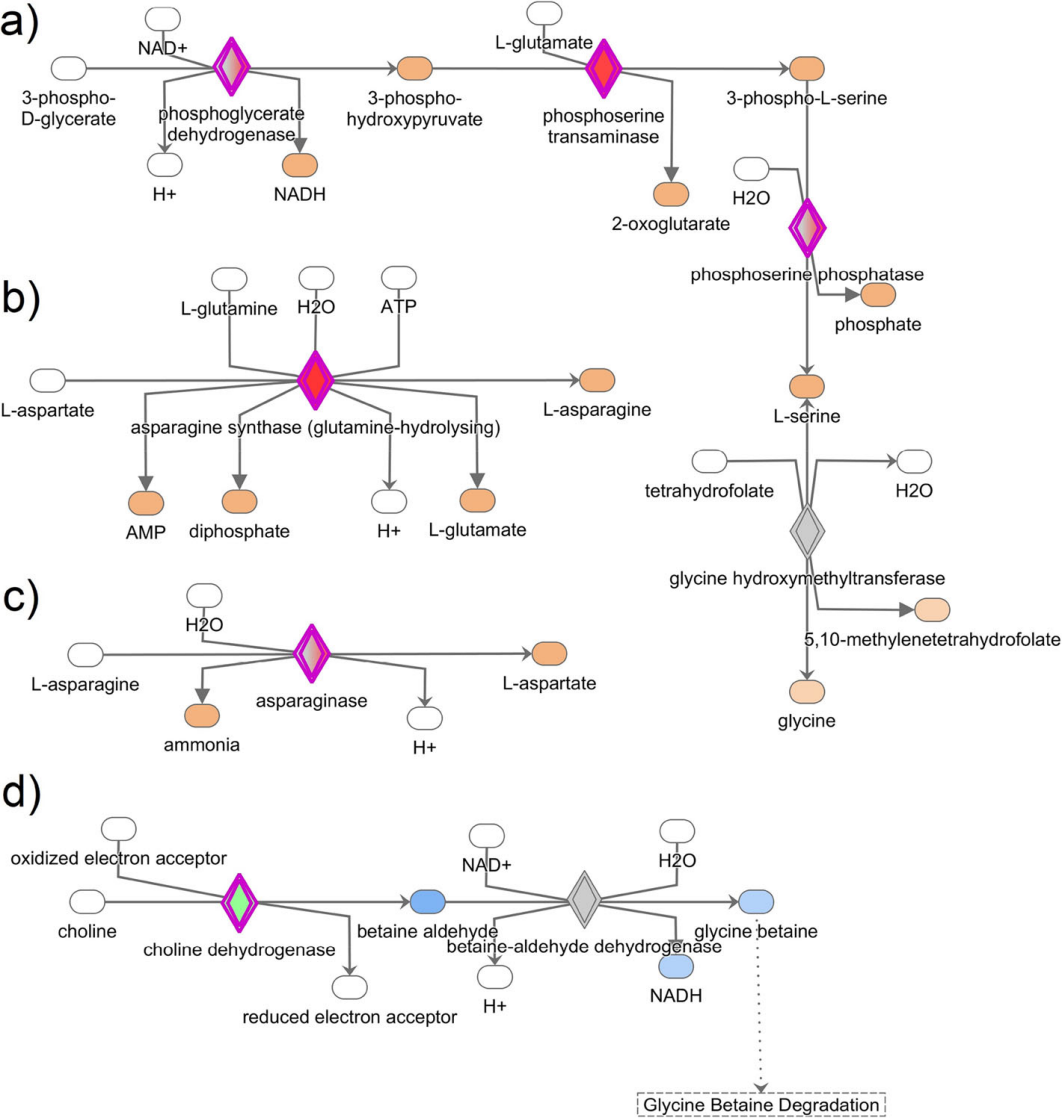
Results of most significant and relevant canonical pathways associated with albumen formation in laying hens. **a** Superpathways of serine and glycine biosynthesis I, **b** asparagine biosynthesis I, **c** asparagine degradation I, and **d** choline degradation. The canonical pathways were analyzed using QIAGEN’s Ingenuity Pathway Analysis (IPA; QIAGEN Inc., https://www.qiagenbioinformatics.com/products/ingenuity-pathway-analysis). Differentially expressed genes in the layers were subjected to IPA analysis, and significant canonical pathways were identified at *p*-value < 0.05. The above-identified canonical pathways demonstrate how the candidate molecules (genes) are involved in amino acid synthesis and degradation

After submitting the DEGs to the ingenuity pathway analysis (IPA), 417 molecules were recognized in its database that belonged to 34 significant canonical pathways (Table 3). Cell cycle control of chromosomal replication, the role of BRCA1 in DNA damage response, mitotic roles of polo-like kinase, cell cycle: DNA damage checkpoint regulation, and role of CHK proteins in cell cycle checkpoint control were the 5 most-significant canonical pathways. Among the significant canonical pathways, 2 pathways (Cell cycle: G2/M DNA damage checkpoint regulation and regulation of cellular mechanisms by calpain protease) were predicted to be activated, while 7 pathways were predicted to be inhibited; the rest lacked sufficient literature to be predicted. We were particularly interested in the most significant metabolic pathways such as serine biosynthesis, superpathways of serine and glycine biosynthesis I, inhibition of matrix metalloproteases, asparagine biosynthesis I, asparagine degradation I, and choline degradation I (Fig. 3) because of their prominent role in albumen synthesis and secretion.

**Table 3:** Significant canonical pathways identified by IPA involved in the egg-white formation

Ingenuity Canonical Pathways	-log (*p*-value)
Cell Cycle Control of Chromosomal Replication	11.2
Role of BRCA1 in DNA Damage Response	7.57
Mitotic Roles of Polo-Like Kinase	6.34
Cell Cycle: G2/M DNA Damage Checkpoint Regulation	5.43
Role of CHK Proteins in Cell Cycle Checkpoint Control	5.05
Hereditary Breast Cancer Signaling	4.68
Pyrimidine Deoxyribonucleotides De Novo Biosynthesis I	4.21
Serine Biosynthesis	4.18
Estrogen-mediated S-phase Entry	4.11
DNA Double-Strand Break Repair by Homologous Recombination	3.95
ATM Signaling	3.85
Superpathways of Serine and Glycine Biosynthesis I	3.64
GADD45 Signaling	3.40
DNA damage-induced 14–3-3σ Signaling	3.40
Regulation of Cellular Mechanics by Calpain Protease	3.13
Antiproliferative Role of TOB in T Cell Signaling	2.86
Neuroprotective Role of THOP1 in Alzheimer’s Disease	2.69
Salvage Pathways of Pyrimidine Ribonucleotides	2.64
Atherosclerosis Signaling	2.52
Cyclins and Cell Cycle Regulation	2.43
Dopamine-DARPP32 Feedback in cAMP Signaling	2.38
Complement System	2.28
Inhibition of Matrix Metalloproteases	2.20
Pyridoxal 5′-phosphate Salvage Pathway	2.10
Hepatic Fibrosis / Hepatic Stellate Cell Activation	2.06
Airway Pathology in Chronic Obstructive Pulmonary Disease	2.03
Breast Cancer Regulation by Stathmin1	1.79
Asparagine Biosynthesis I	1.72
Cell Cycle Regulation by BTG Family Proteins	1.54
Mismatch Repair in Eukaryotes	1.44
Asparagine Degradation I	1.42
Choline Degradation I	1.42
eNOS Signaling	1.37
Cardiac β-adrenergic Signaling	1.32

All the differentially expressed genes in the layers were used in Ingenuity Pathway Analysis and significant canonical pathways based on IPA scores were identified. IPA, ingenuity pathway analysis

### Validation of the expression profiles of selected candidate genes

Following the identification of DEGs, the expression profiles of the 19 most relevant upregulated genes, speculated to be related to the event of albumen synthesis and secretion, were determined in laying, molting, and non-laying hens by real-time PCR (qPCR) assay. The selected candidate genes were *avidin* (*AVD*), *transmembrane protease serine 9* (*TMPRSS9*), *matrix metallopeptidase 1* (*MMP1*), *angiotensin-converting enzyme* (*ACE*), *autophagy-related 10* (*ATG10*), *avian beta-defensin 11* (*AvD11*), *relaxin* (*RLN3*), *matrix metallopeptidase 9* (*MMP9*), *melanotransferrin* (*MELTF*), *glutathione peroxidase 3* (*GPX3*), *cingulin* (*CGN*), *protein C* (*PROC*), *phosphoserine phosphatase* (*PSPH*), *phosphoglycerate dehydrogenase* (*PHGDH*), *asparagine synthetase* (*ASNS*), *phosphoserine aminotransferase 1* (*PSAT1*), *matrix metallopeptidase 10* (*MMP10*), *calpain 2* (*CAPN2*), and *renin* (*REN*).

**Fig. 4:**
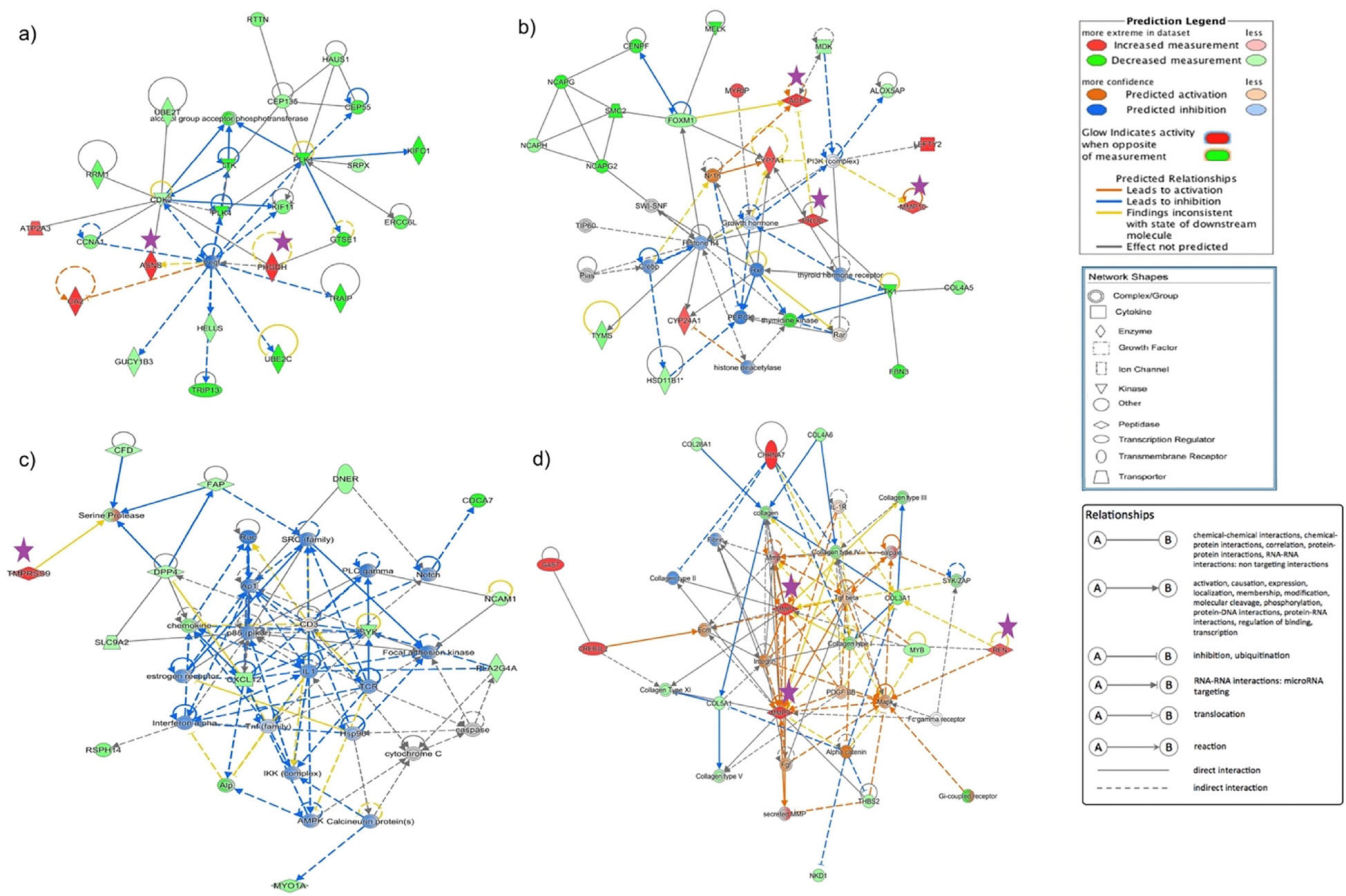
Gene network highlighting some of the candidate genes involved in albumen formation. The candidate genes and their interaction in potentially regulating the synthesis, secretion, and transport of molecules during egg-white formation is shown. This was derived from QIAGEN’s Ingenuity Pathway Analysis (IPA; QIAGEN Inc., https://www.qiagenbioinformatics.com/products/ingenuity-pathway-analysis). **a** PHGDH, ASNS; **b** ACE, MMP10, PROC; **c** TMPRSS9; and **d** MMP1, MMP9, REN. Differentially expressed genes in the layers were used in Ingenuity Pathway Analysis, and significant gene networks based on IPA scores were identified

**Fig. 5:**
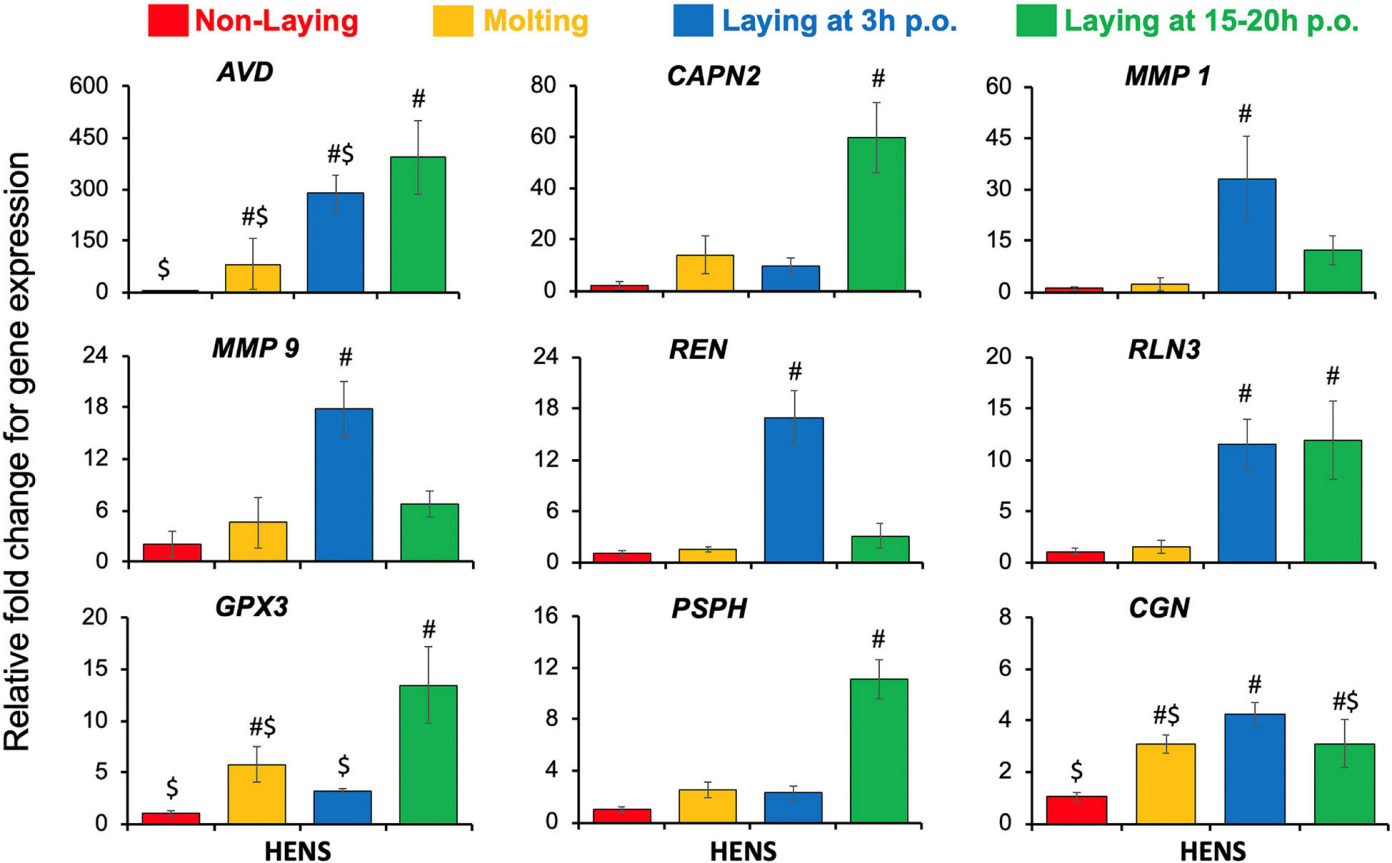
Validation of the gene expression in the magnum of non-laying, molting, and laying hens. Data represented as the mean ± SE. The x-axis represents the physiological status of hens used in the experiment; Y-axis represents relative fold change for gene expression. #, $ denotes significance at *p*-value < 0.05

The gene networks showing the interactions of some selected candidate genes using the IPA network analysis are shown in Fig. 4. For the qPCR gene expression profiles, the double delta Ct (2^-ΔΔCt^) method was used to calculate the relative fold change of the candidate genes after normalization with the house-keeping gene *TATA-Box Binding Protein* (*TBP*). A total of nine genes, amongst the 19 candidate genes, showed significant changes (*p*-value < 0.05) in expression profiles between the experimental groups (Fig. 5). The mRNA expression of *CAPN2* and *PSPH* were highest in laying hens at 15–20 h p.o. compared to either laying hens at 3 h p.o., molting, or non-laying hens. Expressions of *REN*, *MMP1*, and *MMP9* mRNAs were upregulated only in laying hens at 3 h p.o. compared to either laying hens at 15–20 h p.o., molting, or non-laying hens. Expression of *RLN3* gene was increased in laying hens, both at 3 h p.o. and 15–20 h p.o. relative to molting and non-laying hens. *AVD* mRNA was highest in the laying hens at 15–20 h p.o. followed by 3 h p.o. and molting hens, while lowest in the non-laying hens. The expression of *GPX3* mRNA was higher in laying hens at 15–20 h p.o. compared to both non-laying and laying hens at 3 h p.o. The *CGN* mRNA had increased expression in laying hens at 3 h p.o. relative to 15–20 p.o. and molting hens, while significantly higher than non-laying hens. The results obtained from RNA-Seq and qPCR were highly correlated (R^2^ = 0.94; Supplementary Table S4), showing consistency between RNA-Seq and qPCR data for fold change of gene expression.

## Discussion

The magnum is highly glandular tissue, and molecules secreted and/or transported from the luminal and glandular epithelium contribute to the egg albumen. The egg remains in the magnum for 1–3 h to complete the deposition of albumen around the yolk. In the first few hours of the ovulation cycle (1–3 h p.o.), the egg is in the magnum, during which the stored proteins from the magnum epithelium are secreted in the lumen [6]. In the later period of the ovulation cycle (4–23 h p.o.), immediately after the egg has left the magnum, the protein synthesis process begins and continues until the next egg reaches the magnum [6–8]. Using RNA-Sequencing and qPCR, we identified several novel genes and biological pathways associated with egg albumen formation.

**Fig. 6:**
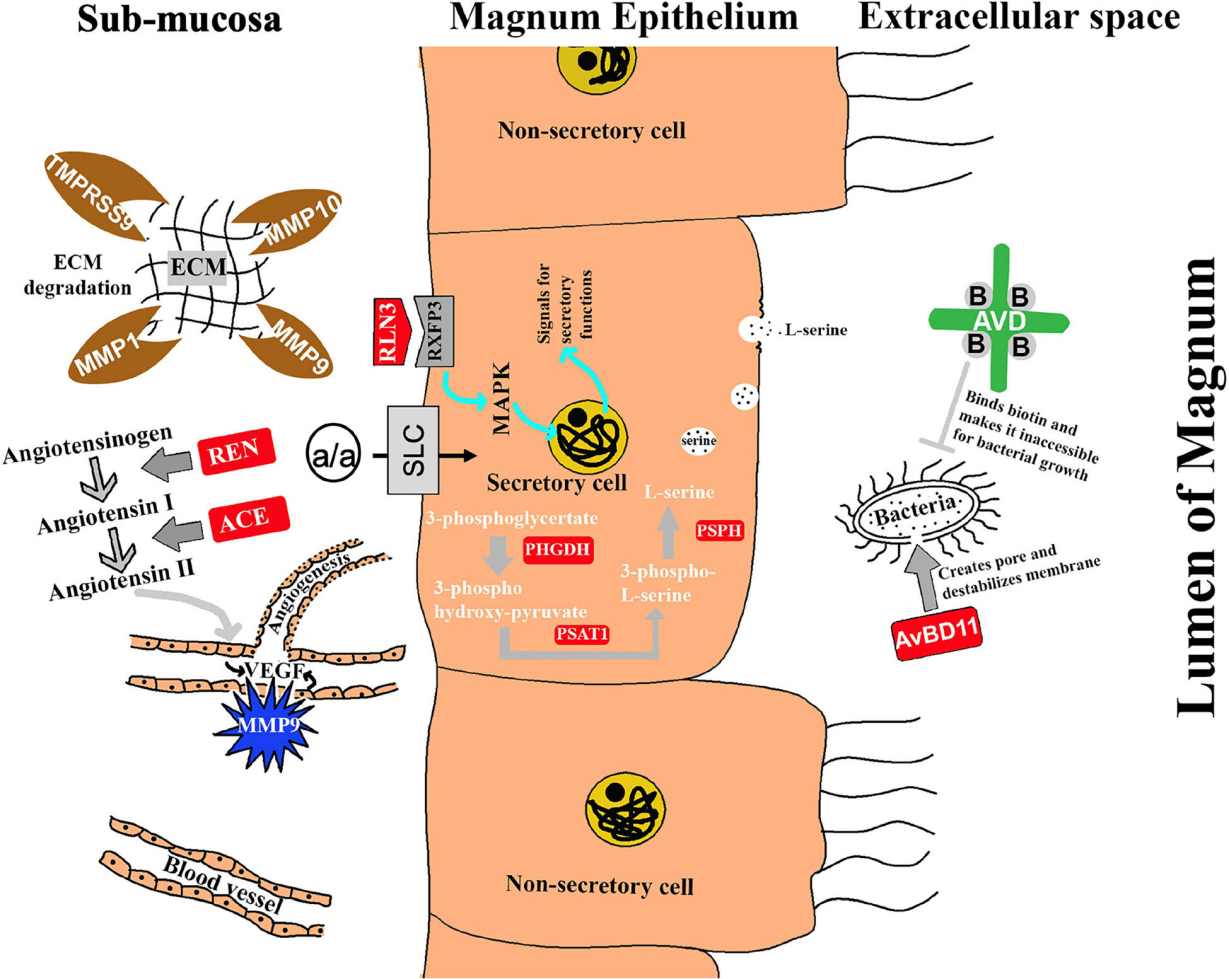
The hypothetical model showing the identified genes and their predicted roles associated with egg-white formation. Solute carriers such as SLC1A4, SLC7A11, SLC7A7, and SLC6A17 may expedite the transport of precursor molecules for protein synthesis. Proteases such as CAPN2, TMPRSS9, MMP1, and MMP9 may be involved in protein maturation and activation, ECM degradation, and angiogenesis for the delivery of molecules from blood circulation so that the magnum epithelium can utilize them for the synthesis of egg-white proteins. Upregulated PHGDH, PSPH, and PSAT1 suggest their active role in the synthesis of amino acids that are basic units of the complex albumen proteins. Increased expression of Relaxin-3 and renin-angiotensin system (REN and ACE) may be linked to their participation in the transport of egg through the oviduct controlling how long the egg stays in the magnum for efficient protein deposition around the yolk. In addition to those genes involved in biosynthesis, some other genes which have a protective function to the egg such as, avidin, avian-beta-defensin 11, and glutathione peroxidase are also incorporated in the egg albumen

In the present study, RNA-Seq data revealed a total of 540 genes differentially expressed between laying (at 3 h p.o.) and non-laying hens. As previously reported, we observed increased expression of genes encoding the common egg-white proteins such as ovalbumin, lysozyme, and avidin in the magnum when the egg was present in this segment of the oviduct [11, 17, 18]. Amongst the DEGs, several proteases (*TMPRSS9*, *ACE*, *REN*, *MMP1*, *MMP9*, *MMP10*, *CAPN2*, and *PROC*) and enzymes for biosynthesis (*PHGDH*, *PSPH*, *PSAT1*, and *ASNS*) were of particular interest. Some of these genes were detected in the microarray and RNA-Seq studies in the magnum [11, 19]. However, their potential role in the formation of egg-white was not reported. In this study, we validated and assessed the specificity of these identified novel genes and pathways in the laying (3 h and 15–20 h p.o), molting, and non-laying hens using qPCR. Then, we used their expression profile to extrapolate their novel role in the synthesis and/or secretion of egg-white proteins based on existing literature. The newly identified genes were involved in antimicrobial defense, matrix remodeling, albumen synthesis and/or secretion, and egg transport (Fig. 6).

### Proteases associated with the albumen synthesis and secretions

Proteases are enzymes having catalytic activity on proteins. There are seven different classes (based on catalytic residue) of proteases, including serine proteases and metalloproteases [20]. Both the serine- and metallo-proteases actively regulate the protein turnover of the extracellular matrix (ECM), influencing various cellular functions [21]. Our RNA-Seq data showed that *TMPRSS9* mRNA was the second most upregulated DEG (FC = 33) in laying hens when the egg was inside the magnum. TMPRSS9, also known as polyserase-I, is a transmembrane type II serine protease that uniquely produces three other proteases, including 2 active ones [22]. TMPRSS9 facilitates the formation of urokinase plasminogen activator that converts plasminogen to plasmin responsible for the degradation of ECM components [23]. Higher expression of *TMPRSS9* uniquely in laying hens suggests that it potentially participates in the degradation of the ECM to release the stored proteins making them available for receptor binding and signaling action, as proposed by ten Dijke et al. [24]. This process is indeed relevant in laying hens when the egg is present in the magnum or the shell gland to keep up with the enormous amount of protein synthesis and secretion.

Matrix metalloproteases (MMPs) are the primary regulators of ECM remodeling. There are 24 members of the MMPs family in vertebrates, including *MMP*-1, − 9, and − 10, which are secreted proteins involved in a wide range of physiological activities such as cellular migration and angiogenesis, and inflammation [25, 26]. In this study, the expressions of *MMP-1*, *MMP-9,* and *MMP-10* were upregulated (FC = 16, 7.5, and 3.5, respectively) in the laying hens with the presence of egg in the magnum, compared to the non-laying hens. MMP1 is down-regulated in magnum when the hen transitions from laying to molting stage [11]. MMP1, also known as collagenase, can degrade the most highly abundant ECM; collagen, in several tissues, including the chicken ovary [27]. MMP-9 (gelatinase) is known to degrade the gelatin matrix [28], provokes angiogenesis [29], and also regulates the laying process in hen [15]. MMP10 breaks down several collagen-related connective tissues [30]. There is no report of *MMP10* mRNA expression in the chicken oviduct; however, a metastatic study has confirmed its association in angiogenesis [31]. Several proteins need to be synthesized and transported into the lumen for deposition around the egg yolk for albumen formation. The required proteins are synthesized in the tubular gland cells of the magnum, which require the rapid transport of amino acids from the blood circulation [6]. We speculate that the higher expression of *MMP-1*, *MMP-9,* and *MMP-10* in the magnum of laying hens are associated with the tissue remodeling and formation of new vasculatures to support the expeditious conveyance of precursor molecules for the biosynthesis of egg-white proteins.

Calpains, on the other hand, are ubiquitous intracellular cysteine proteases having very low specificity for recognition of amino acid sequence. Calpains have a wide range of functions in various tissues, including membrane repair, cell adhesion and motility, cell death, protein cleavage, and activation [32]. Our study reports an increased expression of *CAPN2* in the laying hens during 15–20 h p.o. compared to either molting or non-laying hens. Similarly, the expression of CAPN2 is higher in hens at the laying stage than in the molting stage [11]. Therefore, we posit that CAPN2 is responsible for the maturation and activation of the synthesized egg-white proteins.

Also, we observed that serine protease inhibitor family B member 2 (SERPINB2) was higher (> 3-fold) in the magnum, and similar up-regulation of SERPINB3 expression in the magnum of laying hens was reported by Jeong et al. [11]. Recently, Zhang et al. [33] also reported the upregulation of SERPINF1 and SEPRINH1 when an egg was present in the magnum of duck. This suggests that the SERPIN family of protease inhibitors has an important role in regulating the secretory activity of magnum for egg-white formation. Indeed, proteomic analysis of the egg white has shown that the SERPIN proteins are incorporated in the egg-white [34].

### Transporters of proteins in the magnum epithelium

Cingulin is a protein localized at the tight junction of epithelial and endothelial cells, first discovered in the chicken intestine, and creates a barrier for molecular transport across cells [35]. In the present study, *CGN* mRNA was 5.4 fold higher in laying as compared to the non-laying hens. Cingulin is involved in the organization of the tight junctions, but simultaneously, it inhibits RhoA (Ras homolog gene family member A) activation and suppresses epithelial cell proliferation and gene expression [36]. However, it is also implicated that CGN regulates cell growth and morphology and creates a single layer of small, tightly packed cells [37]. To the best of the available literature on CGN function, we postulate that *CGN* mRNA is involved in the cellular organization and integrity of the magnum epithelium in laying hens regulating molecular transport across the epithelial barrier.

The solute carriers (SLCs) are exclusive membrane transporters that carry several solutes such as amino acids, organic and inorganic ions, and sugars. Several SLC members, including *SLC7A9*, *SLC1A4*, *SLC7A11*, *SLC7A7*, and *SLC6A17,* were increased by 29.9, 5.6, 5.3, 4.5, and 4.4-folds, respectively in the laying hens. The upregulation of these genes in the magnum of laying hens suggests that they actively participate in the transporter of precursor molecules to synthesize egg-white proteins.

### Molecules involved in the biosynthesis

Several enzymes such as *PHDGH*, *PSPH*, *PSAT1*, *ASNS*, *ASPG*, *GALNT6*, *PDE3A*, and *PHYKPL* were increased in laying hens as shown by our RNA-Seq data. GO enrichment analysis revealed that PHDGH, PSPH, PSAT1, and ASNS were involved in amino-acid biosynthesis. The biosynthesis of L-serine from 3-phosphoglyceraldehyde is mediated by three enzymes PHGDH, PSAT1, and PSPH at each successive step, respectively [38]. Interestingly, the mRNA of *PHGDH* had higher expression in laying hens at 15–20 h p.o. (during the albumen synthesis period), and *PSAT1* and *PSPH* mRNAs were also relatively higher in those hens. The upregulated expression of *PHGDH*, *PSAT1*, and *PSPH* in laying hens in this study strongly indicates the biosynthesis of serine in magnum, which may be required to synthesize egg-white proteins. Besides, microarray analysis of the magnum has shown that the expression of ASNS and PSPH is higher at the laying stage compared to the molting stage [11]. A report by Li et al. [39] suggests an additional role of these enzymes (PHGDH, PSAT1, and PSPH) in protection from reactive oxygen species (ROS) by providing the substrate-serine for glutathione synthesis. The antioxidative function of serine biosynthesis enzymes in the magnum is plausible since cells of the magnum are involved in the production of a massive amount of proteins, and concurrently ROS as by-products.

### Genes involved in albumen secretion and/or oviductal transport of egg

Relaxin hormone produced from the ovary and placenta in mammals helps to ease the parturition process by relaxing the ligaments and dilating the cervix. The relaxin-like family peptide has seven peptides, including relaxin-3, which belong to the insulin superfamily. However, a phylogenetic study showed that the chicken genome had lost all the relaxin family peptides, but relaxin-3 having high homology to the human analog [40]. The relaxin-like peptide is produced in granulosa cells of the post-ovulatory follicles, localized in the uterus of laying hens, and influences the oviduct and uterus to aid in oviposition [41]. Also, loss in functionality of this avian relaxin has been shown to cause a drastic delay in oviposition timing [42, 43]. Studies of Brackett [41] and Wilkinson [40] suggest that the hormonal action of relaxin-3 from ovaries help in egg-laying. This study also detected a significant expression of *RLN3* mRNA in the magnum of laying hens (7.5-fold higher) both during albumen synthesis and secretion period. This is a novel report on *RLN3* expression suggesting its synthesis in the magnum, and we hypothesize that its over-expression at 3 h p.o. in the oviduct may be related to the mechanical distention of the magnum to ease the passage of the developing egg and/or secretion of the stored egg-white proteins. Since the mechanical pressure on the walls of the magnum provokes the secretion of the synthesized albumen proteins [9], *RLN3* potentially is one of the markers of mechanical stimulus for the secretion of albumen from the goblet cells of the magnum.

The renin-angiotensin system (RAS), besides its well-known endocrine role in maintaining extracellular fluid in the body, also regulates ovarian growth dynamics [44]. Renin found in ovarian theca cells [45, 46] and angiotensin-converting enzyme (ACE) localized in the granulosa cells and blood vessels of the ovary [47] are the principal components of the RAS system. Apart from the endocrine function of RAS, the localized action of RAS in the ovary is towards follicular development and ovulation [48]. In this study, *REN* mRNA had significantly increased expression in the magnum of laying hens during the albumen secretion period as compared to molting and non-laying hens. The *ACE* mRNA was also higher (14.8 folds) in laying hens relative to non-laying hens. There are some reports on the activity of RAS in the uterus of humans [49], rats [50], rabbits [51], and quail [52]. So far, there is no report on RAS in the chicken oviduct. Verma and Panda [52] reported that ACE is expressed in immature and mature (with exogenous estrogen) quails with the highest expression in magnum, amongst the other oviductal parts. REN and ACE, fundamental molecules of the RAS, are predominantly found in the glandular epithelium of the human uterus, where the RAS had different roles during the menstrual cycle [49]. Collectively, the RAS controls the blood supply to the magnum by altering the vascular smooth muscle tone (through bradykinin), and forming new blood vessels [53]. Also, the RAS system, specifically in the magnum, might aid in relaxing the magnum to retain the egg for sufficient time, allowing optimum deposition of albumen. Concurrently, the expression of the ACE gene in the magnum of pigeon decreases by more than four-fold when the egg has passed through the magnum during the egg-laying cycle [19]. The previous studies in association with the findings of this study suggest that the expression of *REN* and *ACE* in the magnum of laying hens is strong evidence that the RAS system is also involved in the oviductal transport of egg in the chicken.

### Antimicrobials for the egg defense

Antimicrobial agents are crucial for the livability of the hen’s embryo. The albumen holds the yolk (with ovum) in the center of the egg, without any contact with the eggshell. Albumen acts as a thick protective layer consisting of several antibacterial proteins. One such established protein is avidin, and interestingly in our study, *AVD* was the most overly expressed (250.7 folds) mRNA in laying hens. Avidin is also abundant in the egg white [1] and has a very high affinity for biotin required for bacterial growth and proliferation, thus preventing the invasion by microbial pathogens [54].

Another newly discovered and widely studied chicken antimicrobial protein is avian beta-defensins (AvBDs). AvBD11 is among the 14 members of the AvBDs whose mRNA expression was increased by 7.5 folds in the magnum of laying hens in our study. Previous studies have also revealed the expression of *AvBD11* in the egg vitelline membrane, eggshell membrane, eggshells, and magnum, suggesting that the AvBD11 is an important molecule for innate immunity in hens [17, 55–57]. Taken together, AvBD11 incorporation in the albumen protects the developing embryo and might increase the hatchability of the eggs.

### Antioxidant for protection of the magnum epithelium

Glutathione peroxidase (GPX) is a well-known enzyme capable of protecting the cells and tissues from ROS, such as hydrogen peroxidases and other lipid hydroperoxides. GPX3 is an isoform of the enzyme GPX class, localized in plasma and extracellular spaces [58]. We observed a 5.6 folds higher expression of *GPX3* mRNA in the magnum of laying hens during the albumen synthesis period. These findings are indeed concurrent with the underlying physiological activities in laying hens. In the magnum of laying hens, rapid protein synthesis occurs at 4–23 h p.o. indicating that the cells of magnum have increased metabolism. As a result, simultaneous with protein synthesis, there is the release of ROS and other free radicals. So, the increased *GPX3* expression in the magnum is indicative of the protective response against oxidative damage. Also, several other genes differentially expressed in laying hens, such as *urotensin 2* and *spermine oxidase* involved in the production of ROS and hydrogen peroxide [59, 60], respectively, support the fact that oxidative stress is evident in the magnum.

## Conclusion

We have identified a substantial number of novel genes and biological pathways that decipher the cascade of events associated with the albumen formation and deposition in the magnum (Fig. 6). The series of events that occurs in the magnum contributing to the albumen formation include transport of precursor molecules (amino acids, proteins, solutes, and ions), synthesis of proteins (such as ovalbumin, avidin, lysozyme), and secretion or transport of the synthesized proteins to be deposited around the egg yolk. This study revealed the upregulation of several genes in laying hens that are potentially involved in the aforementioned events for egg-white formation (Fig. 6). Solute carriers such as *SLC1A4*, *SLC7A11*, *SLC7A7*, and *SLC6A17* are upregulated in laying hens for expeditious convey of precursor molecules for protein synthesis. Also, the upregulated status of proteases such as *CAPN2*, *TMPRSS9*, *MMP1*, and *MMP9* in laying hens advocates their involvement in protein maturation and activation, ECM degradation, and angiogenesis for the transport of molecules from the blood circulation so that the magnum epithelium can utilize them for the synthesis of egg-white proteins. Increased expression of enzymes such as *PHGDH*, *PSPH*, and *PSAT1* only in laying hens suggests their active role in synthesizing amino acids that are basic units of the complex albumen proteins. During egg formation, laying hens have increased expression of relaxin-3, and renin-angiotensin system (REN and ACE), which posits their participation in the transport of egg through the oviduct controlling how long the egg stays in the magnum for efficient protein deposition around the yolk. They also ease the secretion of albumen from the granular cells for deposition around the egg. In addition to those genes involved in biosynthesis, some other genes have a protective function on the egg, such as *avidin*, *avian-beta-defensin 11*, and *glutathione peroxidase* are also incorporated in the egg albumen. Thus, the findings of this study advanced the knowledge of genes and biological pathways involved in albumen biosynthesis and can potentially be used as markers for formulating strategies to improve the size and quality of the eggs.

## Methods

### Animal husbandry and tissue collection

Hy-Line white (laying, non-laying, and molting) hens were brought from a commercial layer farm (Mikilua Poultry Farm Inc., Hawaii). Before sampling, hens were acclimatized for 2 weeks in the Small Animal Facility of College of Tropical Agriculture and Human Resources, University of Hawaii at Manoa. Hens used for this study were at three different physiological stages; i) laying hens of 35 weeks (*n* = 12), ii) molting hens (*n* = 6) of 60 weeks, and iii) non-laying hens (*n* = 6) between 35 and 60 weeks of age.

The laying hens were in their peak egg production period, while the molting hens were in their first week of programmed molting procedure. The physiological status of molting hens was further verified based on the history of the absence of any laying activity during the experimental period. The molting hens had matured oviduct and ovarian follicular dynamics was evident, but without any follicular clutches or ovulation. The non-laying hens were selected initially based on speculation with the help of flock attendants at the commercial farm. Non-laying hens were identified with meticulous observations and physical assessments such as the shallow abdomen, stiff pubic bones, and dry and puckered cloaca [61]. Therefore, the non-laying hens used for this experiment were identified and selected from different flocks and thus belonged to a range of ages between 35 and 60 weeks. Such non-laying hens were further confirmed based on their atrophied oviduct and absence of any follicular recruitment or maturation in the ovary, examined during necropsy. Each hen was housed in individual pens, reared under a standard light regimen and, fed ad libitum. During the acclimatization period, the egg-laying pattern and time of lay were monitored three times (8 am, 12 pm, and 4 pm) daily for each bird to keep track of its laying performance. To know the exact time of ovulation (~ 30 min after oviposition) for the ease of sampling time points, the hens were monitored hourly from 6 am till 4 pm on the day before sampling.

Hens were euthanized by carbon dioxide asphyxiation. Magnum tissues were collected from laying hens (*n* = 5/group) when the egg was in the magnum (3 h post-ovulation; p.o.) or the uterus (15–20 h p.o.), molting (*n* = 5), and non-laying (*n* = 4) hens. Egg in the magnum/uterus of laying hens was presumed by laying history and confirmed with post-mortem analysis of the oviduct to determine the exact location of egg in the oviductal segment. Magnum tissues were collected from the segments immediately before the site where the egg was present, to prevent any contamination with an excess of albumen from the developing egg. The albumen secretion and deposition from magnum epithelium around the egg yolk starts when the egg is in the magnum, while the secretion of egg-white proteins for the next egg begins once the egg leaves the magnum. Therefore, the expressions of the genes involved in the secretion and synthesis processes are supposed to be upregulated during 3 h p.o. and 4–23 h p.o., respectively. Pieces of magnum tissues were collected, snap-frozen, and stored at − 80 °C until further analysis.

### RNA library preparation and sequencing

Total RNA from the frozen tissues was isolated using TRIzol reagent (Invitrogen, Carlsbad, CA) following standard protocol. The concentrations and quality of the extracted RNA samples were measured using NanoPhotometer® P330 (IMPLEN, Los Angeles, CA) and Agilent 2100 Bioanalyzer (Agilent Technologies, Massy, France), respectively. High-quality RNA samples (RNA integrity number > 8.5) were used for library preparation and sequencing.

RNA-Seq libraries from the magnum tissues of laying (*n* = 3) at 3 h p.o. and non-laying (*n* = 3) hens were prepared using a TruSeq Stranded mRNA kit (Illumina, San Diego, CA) as described previously [62]. Following library preparation, a high sensitivity DNA Bioanalyzer assay (Agilent Technologies, Massy, France) was used to assess the size and quality of the libraries, while KAPA Library Quantification Kit (KAPA Biosystems, Boston, MA) was used to quantify the libraries by qPCR. The sequencing run was executed with a single-end mode with a read length of 1x76bp on a NextSeq 500 (Illumina, San Diego, CA) platform.

### RNA-sequencing analysis

Illumina BaseSpace-created FASTQ files with single-end reads were explored using FastQC (Babraham Institute, Cambridge, UK). Prinseq, a perl script [63] was used to clean the raw reads as mentioned previously [62]. Then, Array Studio (version10; OmicSoft, Cary, NC [64];) was used to align the cleaned against the chicken reference genome Galgal 5.0. The DESeq2 algorithm [65], as implemented in the Array Studio, was used to analyze the differential gene expression in layers with respect to non-layers’ groups. The genes are having a fold change (FC) greater than 3 and Benjamini and Hochberg q-value < 0.05 were categorized as differentially expressed.

### Biological pathways and molecular function analyses

Enriched pathways and molecular function of the upregulated genes in laying hens were determined by using public databases such as the Database for Annotation, Visualization and Integrated Discovery (DAVID [66],) and Kyoto Encyclopedia of Genes and Genomes (KEGG [67],) Pathway as described previously [62]. A list of the upregulated genes was uploaded to the functional annotation tool in the DAVID system, and the chicken was selected as the reference genome for Gene Ontology (GO) enrichment analysis to obtain the enriched biological pathways, molecular function, cellular component, and the pathways. The GO terms with a modified Fisher Exact *p*-value < 0.05 and a threshold gene count of 2 were considered enriched. The Ingenuity Pathway Analysis (QIAGEN Inc. [68],) tool was also employed to gain insights into the molecular networks and canonical pathways of the DE genes. The DE genes were fed to the IPA software, and significant differential analyses were made at a *p*-value < 0.05. Since the IPA is based on the human genome mapping, we tried to derive only credible information as applicable to the hen’s physiology.

### Quantitative real-time RT-PCR (qPCR)

To confirm the accuracy of the results obtained by RNA-Seq, nineteen genes having a predicted function in albumen synthesis and/or secretion were selected for qPCR validation. Primers for qPCR were designed using the NCBI primer blast tool (shown in Supplementary Table S5). Standard qPCR protocols were followed as described by Sah et al. [62] in a reaction mixture of 10 μl. *TATA-Box Binding Protein* (*TBP*) was used as a reference gene after analyzing it along with *glyceraldehyde 3-phosphate dehydrogenase* (*GAPDH*), *beta-actin* (*B-actin*) for stable expression in all the samples. All target genes were analyzed in duplicates, and the expression level was determined using the normalized cycle threshold (Ct) values following the standard curve method. The relative fold change for genes was calculated using the 2^-ΔΔCt^ method and presented as mean ± standard error. Statistical analyses were performed using SAS software (SAS Institute Inc., NC) using a one-way analysis of variance followed by the Tukey-Kramer test to determine significance at *p*-value < 0.05.

## Supplementary Information

**Additional file 1: Table S1.** Filtration and alignment summary of RNA-Seq Reads from magnum in laying and non-laying hens. **Table S2.** Summary of magnum RNA-Seq data mapping to the chicken genome (Galgal5.0). **Table S3**. Differentially expressed genes at FDR_BH < 0.05 and FC > 3 in the magnum of laying and non-laying hens. **Table S4.** Correlation between RNA-seq and qPCR data of relative gene expression in magnum of laying and non-laying hens. **Table S5.** List of primers for the candidate genes used in qPCR assay. Primers for the candidate genes were designed using Primer Blast tool of NCBI with filters of amplicon size between 100 and 250 bp, primers must span an exon-exon junction, melting point between 55 and 60 °C with other filters set at default.

## Data Availability

The datasets generated and/or analyzed for this study are available in the Gene Expression Omnibus (GEO) repository and can be accessed with the Accession Number GSE123588 at https://www.ncbi.nlm.nih.gov/geo/query/acc.cgi?acc=GSE123588. The datasets from Jeong et al. [11] were obtained from 10.1371/journal.pone.0076784.s001 The datasets from Yin et al. [18] are available at PRJNA492958 in NCBI (https://www.ncbi.nlm.nih.gov/bioproject/?term=prjna492958), and was obtained from https://www.sciencedirect.com/science/article/pii/S0888754318305810?via%3Dihub#bi0005 The datasets from Lu et al. [19] does not contain a repository name; however, the data were obtained from mrd23428-sup-0001-APPENDICES.xlsx. The datasets from Zhang et al. [33] are available at GenBank Short Read Archive (SRA: PRJNA493510) and were obtained from https://www.sciencedirect.com/science/article/pii/S0888754320300033#s0075

## Abbreviations

ACE: Angiotensin-converting enzyme
AvD11: Avian beta-defensin 11
ANOVA: One-way analysis of variance
ASNS: Asparagine synthetase
AVD: Avidin
ATG10: Autophagy-related 10
B-actin: Beta-actin
CAPN2: Calpain 2
CGN: Cingulin
DAVID: Database for Annotation, Visualization and Integrated Discovery
DEGs: Differentially expressed genes
ECM: Extracellular matrix
GAPDH: Glyceraldehyde 3-phosphate dehydrogenase
GO: Gene Ontology
GPX3: Glutathione peroxidase 3
IPA: Ingenuity pathway analysis
KEGG: Kyoto Encyclopedia of Genes and Genomes
MMP1: Matrix metallopeptidase 1
MMP9: Matrix metallopeptidase 9
MMP10: Matrix metallopeptidase 10
MELTF: Melanotransferrin
PHGDH: Phosphoglycerate dehydrogenase
PROC: Protein C
PSAT1: Phosphoserine aminotransferase 1
PSPH: Phosphoserine phosphatase
RLN3: Relaxin
REN: Renin
SLCs: Solute carriers
TBP: TATA-Box Binding Protein
TMPRSS9: Transmembrane protease serine 9
